# *SCN4A*-related congenital myopathy in a Han Chinese patient: A case report and literature review

**DOI:** 10.1016/j.heliyon.2023.e23663

**Published:** 2023-12-11

**Authors:** Tina Yee-Ching Chan, Ling-Yin Hung, Tiffany Yan-Lok Lam, Bun Sheng, Frank Ying-Kit Leung, Hencher Han-Chih Lee

**Affiliations:** a Kowloon West Cluster Laboratory Genetic Service, Chemical Pathology Laboratory, Department of Pathology, Princess Margaret Hospital, Hong Kong Special Administrative Region; b Department of Medicine and Geriatrics, Princess Margaret Hospital, Hong Kong Special Administrative Region; c Department of Pathology, Yan Chai Hospital, Hong Kong Special Administrative Region

**Keywords:** SCN4A, Congenital myopathy, Congenital myasthenic syndrome, Channelopathies, Sodium channel

## Abstract

*SCN4A* mutations have been shown to be associated with myotonia, paramyotonia congenita, and periodic paralyses. More recently, loss-of-function variants in the *SCN4A* gene were also noted to be associated with rarer, autosomal recessive forms of congenital myasthenic syndrome and congenital myopathy. Diagnosis is challenging as the initial clinical presentation and histological features on muscle biopsies are non-specific. We report a Han Chinese patient presented with congenital myopathy with two missense *SCN4A* variants. The patient had an antenatal history of reduced fetal movements, polyhydramnios and a very preterm birth. At birth, she was noted to have low Apgar score, respiratory distress syndrome and hypotonia. Delayed motor development was noted in early childhood. Dysmorphic features such as an elongated face, dolichocephaly and high arched palate were present. At 16 years of age, the patient developed progressive muscle weakness and was wheelchair-bound by age 20. Muscle biopsy revealed non-specific changes only. Targeted hereditary myopathy panel testing by next generation sequencing revealed two previously unreported missense variants c.1841A > T p.(Asn614Ile) and c.4420G > A p.(Ala1474Thr) in the *SCN4A* gene. The clinical features of *SCN4A*-related congenital myopathy and myasthenic syndrome were reviewed. This case exemplifies the utility of next generation sequencing in the diagnosis of undifferentiated muscle disease.

## 1 Introduction

The voltage-gated sodium channels are integral cell membrane proteins responsible for cell depolarization and propagation of action potentials in nerves and muscles. The alpha subunit of the channel is comprised of four homologous domains, each with six transmembrane helices which form the pore for sodium ion passage [1]. These voltage-gated sodium channels are encoded by at least nine different isoforms in different tissues. *SCN4A* (MIM *603967) encodes the isoform Na_V_1.4 which is highly expressed in skeletal muscle. The Na_V_1.4 channelopathies encompass several phenotypes, including the autosomal dominant inherited disorders of sodium channel myotonia congenita, atypical, acetazolamide-responsive (MIM #608390), paramyotonia congenita (MIM #168300), hypokalemic periodic paralysis (MIM #613345) and hyperkalemic periodic paralysis (MIM #170500) which are caused by gain-of-function mutations in *SCN4A*. On the contrary, patients with loss-of-function *SCN4A* variants present with autosomal recessive phenotypes including congenital myasthenic syndrome 16 (MIM #614198) [[2], [3], [4], [5], [6], [7]] and congenital myopathy 22A and 22B (MIM #620351 and MIM #620369) [[7], [8], [9], [10], [11], [12], [13], [14]]. The recessive phenotypes are rare and the clinicopathologic features and treatment modalities are not well described in the literature compared to the dominant phenotypes. For instance, patients with the dominantly inherited hypokalaemic or hyperkalaemic periodic paralysis (PP), usually develop episodic paralytic attacks associated with abnormal blood potassium levels, with well-characterized triggers such as vigorous exercise or having a too high or too low carbohydrate intake [15]. Development of a fixed permanent myopathy has also been reported in some patients [16]. Sodium channel myotonia and paramyotonia congenital are characterized by inability of muscle to relax after voluntary effort, which are respectively improved and worsened with repeated muscle effort. Needle electromyography showed characteristic myotonic discharges that are evoked by needle insertion or with voluntary contraction. Functional analyses of the mutant sodium channels reported in these autosomal dominant phenotypes invariably revealed functional changes associated with gain-of-function changes, such as abnormal gating current in the case of hypokalaemic PP, or impairment of fast and slow inactivation and enhanced activation of the mutant channel in the other disorders [17]. Congenital myasthenic syndromes (CMS) are a heterogenous group of disorders characterized by defective signalling at the neuromuscular junction that arises from germline pathogenic variants [18]. The hallmark of these disorders is also muscle weakness, which is usually worse upon exertion. The age of onset, severity of symptoms, distribution of muscle weakness and other concomitant features can vary greatly amongst patients. Congenital myopathy (CM) is a broad term that is mainly used for rare, inherited, primary muscle disorders [19]. Typically individuals usually present at birth or in infancy with hypotonia, weakness, hypoactive deep tendon reflexes, and delayed motor milestones. Although it is worthwhile to note that some patients may present later in life during infancy or childhood. The muscle weakness may slowly progress over time. In addition, prominent facial weakness and ptosis are often present, together with dysmorphic features such as an elongated face, dolichocephaly, and a high-arched palate. Due to the non-specific, overlapping and myriad presentations, it is sometimes difficult to differentiate between these various entities by clinical and histopathological features and to arrive at a definitive diagnosis. Functional studies of the mutant sodium channels in the recessive phenotypes often show loss-of-function defects [2,3], such as hyperpolarizing shift of voltage dependence of fast inactivation, slower recovery from inactivation and faster onset of slow inactivation [4]. Here we report the first case of *SCN4A*-associated congenital myopathy in a Han Chinese patient with two novel mutations. The cosegregation pattern suggested these mutations to be associated with loss-of-function. The clinicopathologic features and genetic findings in the present case are described and compared to reported cases in the literature.

## 2 Case report

Patient and methods.

### 2.1 Patient

**Fig. 1:**
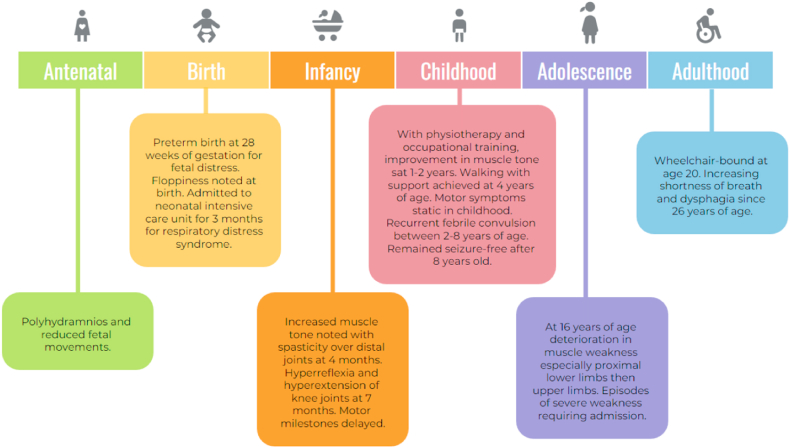
Chronological summary of the patient's onset and development of myopathy.

A 32-year-old Chinese female was referred to our Neurology Clinic for progressive weakness. Fig. 1 summarises the patient's clinical manifestations of myopathy. She was born of non-consanguineous parents by Caesarean section at 28 weeks for fetal distress. Antenatal period was remarkable for reduced fetal movements and polyhydramnios. At birth, she was noted to be floppy. The Apgar score was five at 1 min and seven at 5 min. She had a very low birth weight of 0.915 kg. She was admitted to the neonatal intensive care unit for respiratory distress syndrome requiring neonatal nasal intermittent positive pressure ventilation for three months. Poor feeding and a palatal groove were also noted. At four months of age, she received physiotherapy for generalized hypotonia especially over trunk and proximal joints; however, an increased muscle tone was noted on exertion with signs of spasticity over distal joints. She was diagnosed to have cerebral palsy. At seven months of age, hyperreflexia and hyperextension of the knee joints were noted. There was poor exercise endurance and she was easily fatigued. A delayed gross motor development was observed: the presence of severe head lag at seven months; sit with support at 11 months; sit without support at 20 months; walk with support at four years of age. The patient received intensive physiotherapy and occupational training from four months of age; a clinical improvement of her muscle tone and motor symptoms was noted at one to two years of age. Despite this, mild muscle wasting was apparent from five and a half years of age; with myopathic facial features such as triangular mouth and ptosis noted during physical examination at six years old. Thereafter, her motor symptoms remain static during childhood.

The patient had recurrent febrile convulsions from two to eight years of age; the seizure was controlled with anticonvulsants and the patient remained seizure-free from the age of eight. Dysarthria was also noted and the patient received speech therapy. At 16 years of age, the patient noted a deterioration of her muscle weakness: the weakness first affected bilateral lower limbs then progressed to involve upper limb muscles, with the proximal muscle groups being affected more than the distal ones. The muscle weakness was especially prominent early in the morning upon awakening. Also, the patient reported fatigability and myalgia on exertion. There were occasional episodes of severe weakness lasting hours to days requiring admission, which were sometimes preceded by exercise and a high carbohydrate meal; but the patient was not aware of any definite reproducible triggers. By the age of 20, the patient became wheelchair-bound. Increasing shortness of breath and dysphagia were reported at the age of 26. There was no significant family history of neuromuscular disorders, including that of the patients’ parents and younger sister.

**Fig. 2:**
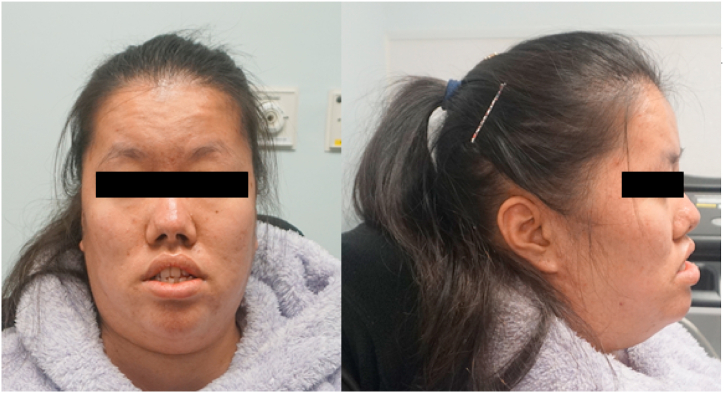
Clinical photos of the patients showing soft dysmorphic features such as elongated face, dolichocephaly and high arched palate.

Physical examination showed a high-arched palate, an elongated face with dolichocephaly (Fig. 2). Muscle power assessment with Medical Research Council (MRC) scale showed a power of grade 3- and 2 over the proximal muscles in the upper and lower limbs respectively. A muscle power of MRC grade 3+ over the distal muscle groups for both upper and lower limbs was also noted. Deep tendon reflexes, sensory and cerebellar examinations were normal. Tensilon test was performed on both upper and lower limbs and the result was negative.

**Fig. 3:**
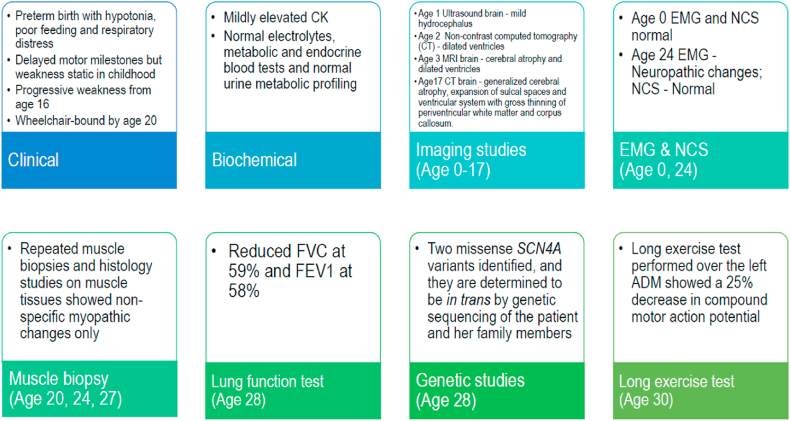
Timeline of the major diagnostic investigations performed.

As the new progression of muscle symptoms at the age of 20 were atypical of cerebral palsy, which is expected to follow a non-progressive course, extensive investigations were made for our patient after this new onset of symptoms, and their findings were summarized in Fig. 3. Biochemical tests on blood specimens revealed a mildly elevated plasma creatine kinase (CK) up to 379 U/L. There was no documented hypokalaemia during her admissions for severe weakness. Thyroid-stimulating hormone, free thyroxine, lactate and ammonia levels were normal. Anti-acetylcholine receptor antibody was negative. Plasma acylcarnitine and amino acids profile, as well as urine metabolic profiling with LC-MS/MS for organic acids, purines and pyrimidines, amino acids and sugars showed no pathological patterns. A dried blood spot test for Pompe disease assaying whole blood acid alpha-glucosidase activity was normal.

Magnetic resonance imaging (MRI) of the brain at three years of age showed generalized cerebral atrophy and dilated ventricles. At the age of 17, computed tomography (CT) of the brain revealed generalized cerebral atrophy, expansion of sulcal spaces and ventricular system with gross thinning of periventricular white matter and corpus callosum. Features suggestive of arrested hydrocephalus or normal pressure hydrocephalus was also noted. No significant interval changes were observed. Findings were compatible with perinatal hypoxic insult.

**Fig. 4:**
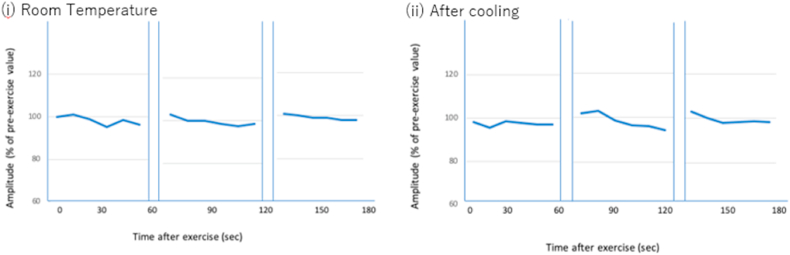
Percentage change in compound motor action potential on short exercise test at room temperature (i) and after cooling (ii).

**Fig. 5:**
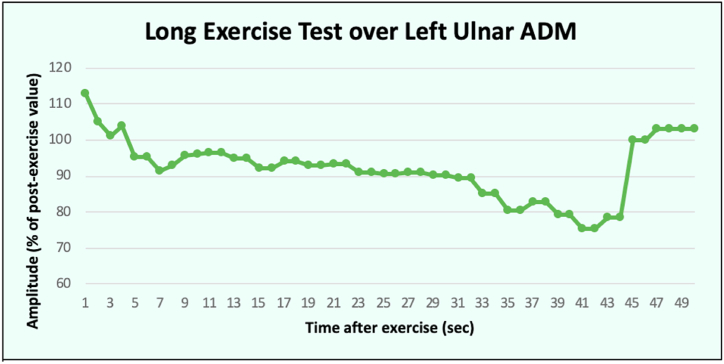
Percentage change in compound motor action potential on long exercise test.

Motor and sensory nerve conductions were normal. Electromyography (EMG) was normal at rest with no electrical myotonia or spontaneous activity observed. Giant motor unit potential (MUP) and neuropathic changes were noted in both proximal and distal muscle groups. A short exercise test was performed over the right abductor digit minimi (ADM). After ten seconds of sustained exercise, baseline supramaximal compound muscle action potential (CMAP) was recorded, and CMAP was recorded following every 10 seconds for 60 seconds in total. There were no significant post-exercise CMAP changes after successive three trials (Fig. 4i) and limb cooling (Fig. 4ii). A long exercise test was performed at 30 years of age over the left ADM. After five minutes of sustained exercise, supramaximal CMAP responses were recorded before exercise, and every 2 min after exercise for 50 minutes in total. Maximal decrement (25 %) was observed around 45 minutes post-exercise (Fig. 5), the magnitude of which was greater than the reported limits of normal (−20 to +10 %) but below the usual magnitude of change (−41 to −61 %) observed in periodic paralysis [20].

A pulmonary function test performed at 28 years of age for increasing shortness of breath showed a reduction in both forced vital capacity (FVC) at 59 % and forced expiratory volume (FEV1) at 58 %.

### 2.2 Muscle biopsy

Muscle biopsies were received fresh for frozen sections and enzymatic stains while parts of the specimens were formalin-fixed and paraffin-embedded for histology and glutaraldehyde-fixed and resin-embedded for transmission electron microscopy. Frozen sections were stained with hematoxylin and eosin (H&E), oil red O (ORO), Periodic acid–Schiff (PAS) stain, oxidative enzymes (reduced nicotinamide adenine dinucleotide-tetrazolium reductase (NADH-TR); succinate dehydrogenase (SDH); cytochrome C oxidase (COX) and modified Gomori trichrome (GT) methods.

### 2.3 Molecular genetic testing

Genomic DNA was extracted from ethylene diaminetetra-acetic acid (EDTA) white blood cells in whole blood samples collected from the proband and her family members using a QIAamp blood mini kit (Qiagen, Hilden, Germany). Next-generation sequencing was performed for the proband using targeted gene capture on Illumina MiSeq Sequencing System with regions of interest restricted to the coding regions and the 10-bp flanking regions of a selected panel with 83 myopathy genes (genes listed in Supplementary Table 1). Alignments to GRCh37/hg19 human genome assembly ad variant calls were generated using NextGENe 2.4.2 (SoftGenetics, State College, PA). Variants identified were annotated and analysed with VariantStudio 3.0.12 (Illumina, San Diego, CA). Genbank accession number NM_000334.4 and NP_000325.4 were used for *SCN4A*. Orthologue alignment was retrieved from Alamut Visual version 2.8 (Interactive Biosoftware, Rouen, France) while paralogue alignment with other sodium channels including *SCN1A*, *SCN2A*, *SCN3A*, *SCN5A*, *SCN7A*, *SCN8A* and *SCN9A* was performed using Kalign multiple sequence alignment algorithm [21].

## 3 Results

### 3.1 Muscle biopsy

**Fig. 6:**
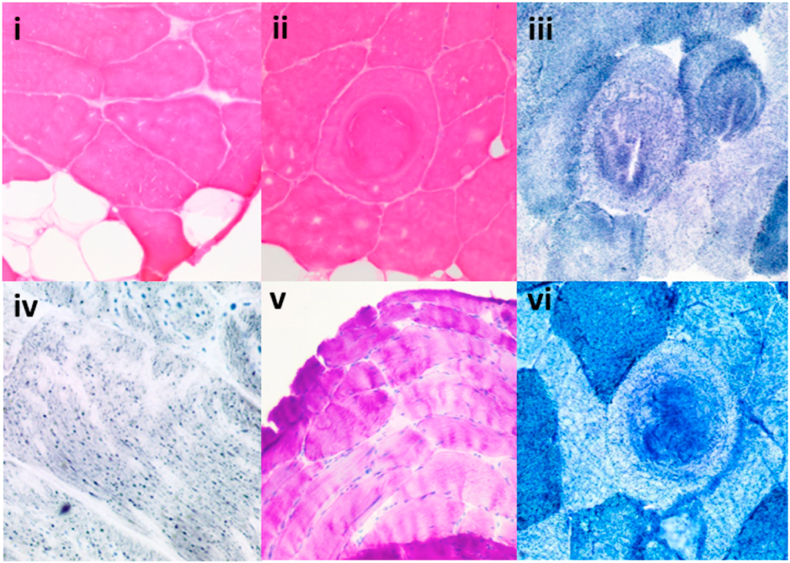
Muscle biopsy (*intended for colour reproduction) Non-specific changes seen in muscle biopsies. H&E staining showing fatty infiltration and mild fibre size variation (i), whorled fibres and internalized nuclei (ii) NADH-TR staining showed whorled fibres (iii) Sudan black staining showed scattered lipid droplets (iv). PAS staining showed normal glycogen content (v) PFK staining showed preserved phosphofructokinase content (vi). H&E: Hematoxylin & Eosin; NADH-TR: reduced Nicotinamide adenine dinucleotide tetrazolium reductase; PAS: Periodic acid-Schiff; PFK: Phosphofructokinase. (For interpretation of the references to colour in this figure legend, the reader is referred to the Web version of this article.)

Repeated muscle biopsies over quadriceps, deltoid and right thigh were performed at the age of 20, 24 and 27 respectively. H&E staining showed fatty infiltration and mild fibre size variation (Fig. 6i), whorled fibres and internalized nuclei (Fig. 6ii). There were no fibre grouping, inflammatory changes or perifasicular atrophy. NADH-TR staining showed whorled fibres (Fig. 6iii). Sudan black staining on biopsy taken in 2011 showed scattered lipid droplets (Fig. 6iv), which was not evident in repeated biopsy in 2018. PAS staining showed normal glycogen content (Fig. 6v) while enzyme histochemical staining showed preserved phosphofructokinase content (Fig. 6vi). Further ultrastructural examination in 2018 showed occasional myofibrillary disarray and abnormal mitochondria which appeared enlarged, elongated to rectangular shaped, and with the presence of paracrystalline “parking lot” inclusions.

### 3.2 Molecular genetic analyses

**Fig. 7:**
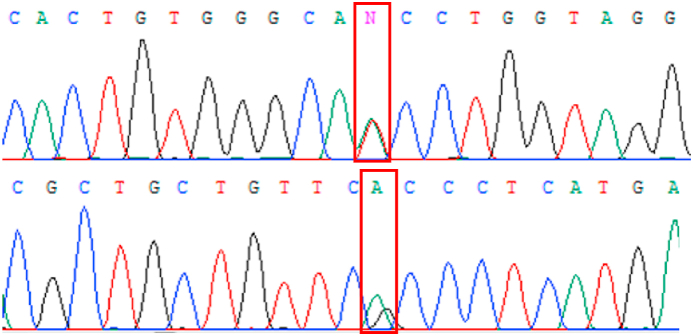
Electropherogram showing two heterozygous missense variants in the *SCN4A* gene.

Next-generation sequencing with targeted panel revealed the patient was heterozygous for two previously unreported missense variants in the *SCN4A* gene: NM_000334.4:c.1841A > T p.(Asn614Ile) and NM_000334.4:c.4420G > A p.(Ala1474Thr) (Fig. 7). No pathogenic variants were detected in other genes of interest. Analysis of asymptomatic family members confirmed the compound heterozygosity of the two variants; the father and younger sister of the patient were heterozygous for the c.4420G > A p.(Ala1474Thr) variant while the mother was heterozygous for the c.1841A > T p.(Asn614Ile) variant.

### 3.3 Management and outcome

As currently there are no curative therapies available, the patient was managed with supportive treatment, including physiotherapy, respiratory exercises and symptomatic medications. Despite this, the patient reported increased myalgia and fatigue at the age of 32; she also reported increase in choking and is currently receiving speech therapy for swallowing training.

## 4 Discussion

We report here a 32-year-old Chinese patient compound heterozygous for two previously unreported missense variants c.1841A > T p.(Asn614Ile) and c.4420G > A p.(Ala1474Thr) in *SCN4A*.

**Fig. 8:**
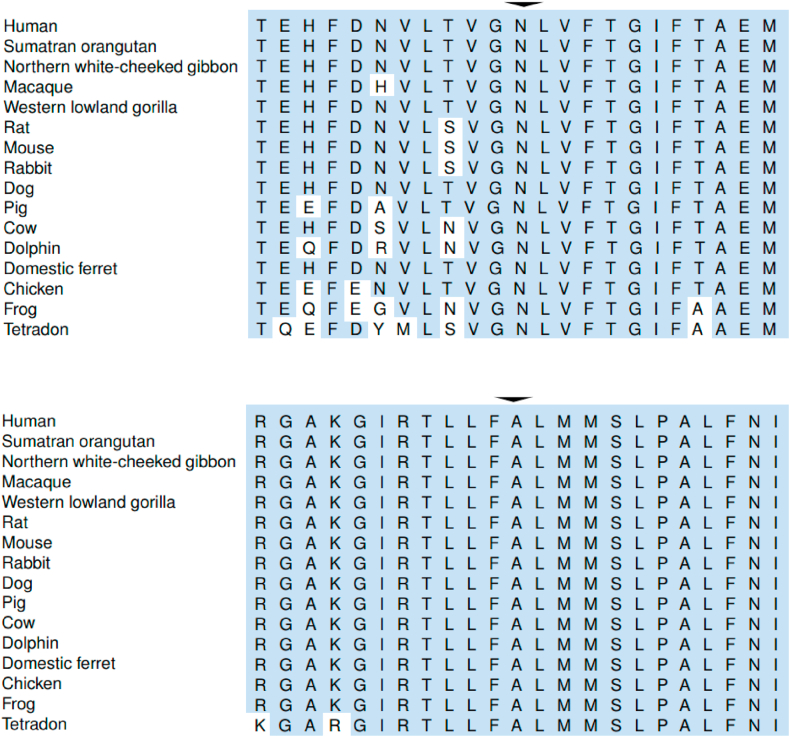
Affected residues are highly conserved across orthologues. Alignment to orthologues with sequences retrieved from Ensembl Compara (with Alamut Visual). Upper panel: Asparagine at residue 614 (arrowhead). Lower panel: Alanine at residue 1474 (arrowhead). Conserved residues are indicated in shaded boxes.

**Fig. 9:**
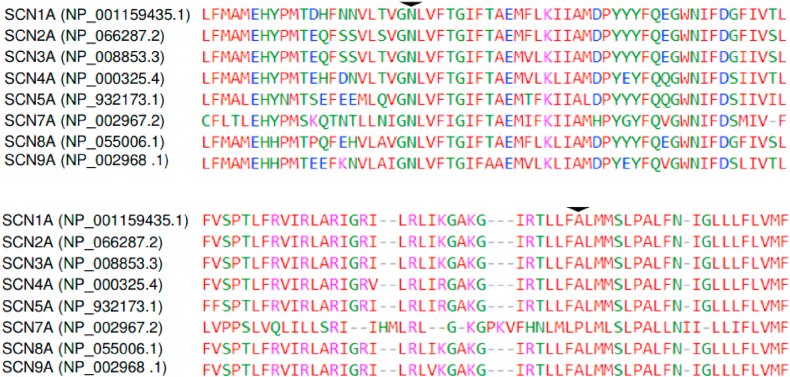
Affected residues are highly conserved across paralogues with Kalign alignment. Upper panel: Asparagine at residue 614 (arrowhead). Lower panel: Alanine at residue 1474 (arrowhead).

The c.1841A > T p.(Asn614Ile) variant is located at the S2 helix in the transmembrane domain II of Na_V_1.4. The residue Asn614 is found at the second last residue of exon 11. It is highly conserved across orthologues and paralogues (Fig. 8, Fig. 9). The variant was absent from control subjects in the Exome Sequencing Project, 1000 Genomes Project and Genome Aggregation Database. Functional study data was not available and the variant had not been reported in the literature in individuals with *SCN4A*-related conditions. In silico analyses by MetaSVM [22], PROVEAN [23] and SIFT [24] predicted the variant to be damaging while PolyPhen-2 [25] predicted the variant to be benign. Asn614 with polar side chain is located at an extracellular negative charge cluster critical for open-state stability of voltage-gated ion channels in skeletal sodium channel isoform Na_V_1.4 [26]. An introduction of a basic side chain (lysine) at this residue (Asn614Lys; N614K) of human skeletal muscle sodium channel isoform Na_V_1.4 by site-directed mutagenesis results in a right-shifted conductance-voltage (G-V) relationships [27]. Furthermore, Pless et al. showed that the substitution of the Asn614 residue with an aspartic acid with a negative side chain increase open state stability; the Asn614Asp mutant showed a left-shift in the G-V [28]. In our patient, the variant p.(Asn614Ile) substitutes an asparagine with the smaller and more hydrophobic isoleucine. We postulated that the introduction of a hydrophobic side chain in this critical, highly conserved residue Asn614 will impact the open-state stability significantly. Based on three-dimensional in silico prediction tool Have (y)Our Protein Explained (HOPE), the variant is predicted to affect the channel interaction with the membrane lipid [29].

The residue Ala 1474 is located at cytoplasmic loop between the S4 and S5 helices in domain IV. It is conserved across orthologues and paralogues (Fig. 8, Fig. 9). The variant is present at a very low allele frequency of 0.0437 % (8 out of 18328 chromosomes) among East Asians in the Genome Aggregation Database. Functional study data for this variant was not available at the time of reporting. In silico analyses by MetaSVM, PROVEAN and SIFT predicted the variant to be damaging while PolyPhen-2 predicted the variant to be possibly damaging. The c.4420G > A p.(Ala1474Thr) variant substitutes an alanine with a hydrophobic side chain with threonine containing a polar uncharged side chain. The S4-5 interhelical region was known to consist of a highly conserved group of small hydrophobic residues playing a critical role in sodium channel activation-inactivation coupling [30]. Based on HOPE prediction, the larger size of threonine may not be able to fit in the protein core and may affect the hydrophobic interactions [29].

In our patient, the segregation pattern of this family, the normal plasma potassium levels during the proband's repeated admissions for severe weakness, the lack of clinical and electrophysiological evidence of myotonia, and the high penetrance of some of the dominant *SCN4A* disorders [31], suggest that the residues involved could be associated with loss-of-function phenotypes, although functional studies are needed for further evaluation.

Including our proband, a total of 27 individuals from 18 families with *SCN4A*-associated myasthenic syndrome and myopathy have been reported as of February 2023 (Supplementary Table 2). Among these patients, antenatal complications were frequent, including polyhydramnios (11/27), limb contractures (6/27), hydrops (6/27), preterm birth (4/27) [10] and breech presentation (3/27) [5,7,8]. Seven patients died in utero or shortly after birth [8]; In the remaining 20 patients, weakness was neonatal in onset (18/20), except for two cases with onset reported in childhood [11] and teenage [3] respectively. In the 18 patients with neonatal-onset disease, respiratory distress and difficulty with sucking and swallowing were common (11/18); They were noted to be episodic or fluctuating in four cases [2,5,6,8] and showed gradual improvement throughout childhood in three cases [8,11]. In childhood and adulthood, weakness mainly involved ocular, bulbar, axial and proximal limb muscles with prominent fatigability and myalgia. Half of the patients (10/20) reported episodic weakness and in three subjects, an association with febrile illness or exercise was noted [8,9]. On physical examination, dysmorphic features including elongated face/dolichocephaly (9/20) and high-arched palate (9/20) were most frequently reported.

Intriguingly, our patient had experienced a period of static neurology during her childhood before worsening of her motor symptoms as she approaches adulthood. This seemingly stepwise progression of muscle weakness had also been observed in two other cases of congenital myopathy [4,8] and one case of congenital myasthenic syndrome with childhood onset of disease [3]. Some patients with congenital myopathy even experienced improvement in motor symptoms during childhood [8,11]. It is uncertain the exact mechanism underlying this pattern of disease progression, and whether the residual function in the mutant *SCN4A* alleles, other genetic factors e.g. compensatory mechanisms from other ion channels, epigenetic mechanisms or environmental factors play a role.

Biochemical findings, including plasma potassium levels, were unremarkable in most subjects except a mildly raised creatine kinase (3/13). Nerve conduction and electromyography may be completely normal (5/12) or may demonstrate a myopathic pattern (1/12), myotonic discharges (1/12) or decremental response on repetitive nerve stimulation (4/12). Decremental response was noted in three cases on long exercise testing, but the magnitude of decrease (between 25 % and 40 %) was below that observed in periodic paralysis or non-dystrophic myotonic disorders. Out of the five patients with magnetic resonance imaging of the muscles performed, selective fat replacement of gluteus maximus, sartorius, adductor magnus and soleus muscles were noted in four patients [8,9,11]. For muscle histology, findings described include non-specific myopathic changes, fatty infiltration, endomysial fibrosis [8,9,11,14], and subsarcolemmal collection of mitochondria with abnormal size and shape [10]. A distinctive pattern of a crown-like arrangement of nuclei around a central core-like structure was observed in only one case [9]. Overall, the clinical, biochemical, electrophysiological and histological features of the phenotype are heterogeneous and non-specific, which can lead to significant difficulties and delays in diagnosis for these patients.

**Fig. 10:**
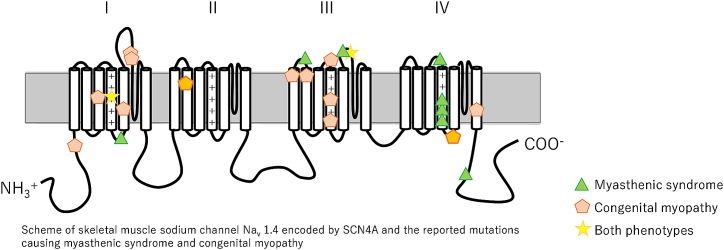
Sodium Channel. Illustration showing a scheme of skeletal muscle sodium channel encoded by SCN4A. Known pathogenic variants causing myasthenic syndrome were indicated using a green triangle, while variants causing congenital myopathy were indicated using a pentagon. Variants reported to cause both conditions were indicated with a star. (For interpretation of the references to colour in this figure legend, the reader is referred to the Web version of this article.)

Unlike in hypokalaemic periodic paralysis in which disease-causing variants typically affect the outermost arginines in the voltage-sensitive S4 segment of the channel [32], there is no clear genotype-phenotype relationship observed in the recessive phenotypes. Of the 26 reported mutations causing the recessive phenotypes, four are null variants (frameshift, nonsense or splicing variants), and are reported in patients presenting with either myasthenic or myopathic phenotypes. The remaining variants are missense variants and are randomly distributed along the channel (Fig. 10).

Use of various pharmacotherapy have been reported in the attempt to alleviate the motor symptoms, especially in the myasthenic patients. They include acetazolamide [4,6,7], pyridostigmine [2,6,7], salbutamol [7] and amifampridine [6], but none of these agents appear to be universally effective. This could also be explained by the heterogeneous nature of mutations involved with variable mechanisms leading to the loss of channel function.

One limitation of our study is the lack of functional studies. However, our findings are supported by in silico analyses across various platforms that predict the pathogenicity of the variant in question. Another limitation is that our patient had accompanying cerebral palsy and childhood epilepsy, which complicates the clinical presentation and may make it more difficult to conclude the direct causality of the *SCN4A* variants on some of the clinical presentations.

To overcome the limitations of existing modalities (e.g. neurophysiological studies and muscle biopsy) used in the diagnosis for various muscular disorders, our laboratory has previously developed and validated the Flexi-Myo panel approach for patients with suspected muscle disorders [33]. Our case reiterates the value of using next generation sequencing in diagnosing rare genetic causes of myopathy, especially when non-specific muscle biopsy findings are encountered. In our patient, for instance, three muscle biopsies were taken from different sites in eight years before a definitive diagnosis was made.

In conclusion, we have reported a case of *SCN4A*-associated congenital myopathy in a Han Chinese patient. It is inherited in an autosomal recessive pattern and the clinical phenotype was associated with two putative loss-of-function variants. Twenty-seven reported cases of recessively-inherited congenital myopathy/myasthenic syndrome in literature were also reviewed. The phenotypes associated with *SCN4A* loss-of-function mutations are likely under-reported and under-diagnosed owing to the non-specific clinical, biochemical and histological findings. Panel testing may be advantageous to uncover more cases of *SCN4A*-related myasthenia syndromes or myopathy and to gain further insight into the pathogenesis and uncover effective treatment modalities for these patients.

## 5 Compliance with ethical standards

Written informed consent was obtained from the patient. Approval for the study was obtained from Kowloon West Cluster Clinical Research Ethics Committee (Ethics approval number KW-EX-09-155). A separate Author Declaration form was duly signed and submitted together with this manuscript. The signed, original informed consent was retained and stored according to institutional guidelines and it is available on request.

## Funding

This research received no specific grant from any funding agency in the public, commercial, or not-for-profit sectors.

## Data availability statement

Data supporting this study are openly available from the LOVD v.3 database with the accession Variant ID 0000920840 for NM_000334.4:c.1841A > T and Variant ID 0000920841 for NM_000334.4:c.4420G > A.

## CRediT authorship contribution statement

**Tina Yee-Ching Chan:** Conceptualization, Data curation, Formal analysis, Investigation, Methodology, Project administration, Resources, Software, Validation, Visualization, Writing – original draft, Writing – review & editing. **Ling-Yin Hung:** Conceptualization, Data curation, Formal analysis, Investigation, Methodology, Project administration, Resources, Software, Supervision, Validation, Visualization, Writing – original draft, Writing – review & editing. **Tiffany Yan-Lok Lam:** Conceptualization, Data curation, Formal analysis, Investigation, Resources, Writing – original draft. **Bun Sheng:** Conceptualization, Data curation, Investigation, Project administration, Supervision. **Frank Ying-Kit Leung:** Conceptualization, Data curation, Formal analysis, Investigation, Methodology, Resources, Software, Supervision, Writing – original draft. **Hencher Han-Chih Lee:** Conceptualization, Data curation, Formal analysis, Investigation, Methodology, Project administration, Resources, Software, Supervision, Writing – original draft.

## Declaration of competing interest

The authors declare that they have no known competing financial interests or personal relationships that could have appeared to influence the work reported in this paper.
