# Supplementary material for: SCN4A-related congenital myopathy in a Han Chinese patient: A case report and literature review
Source: Heliyon. 2023 Dec 11;10(1):e23663. doi: 10.1016/j.heliyon.2023.e23663 (PMC10770507; doi:10.1016/j.heliyon.2023.e23663)
Supplement: Multimedia component 1 [file mmc1.docx]

**Supplementary Table 1**

The genes included in the congenital myopathy panel are as follows:

*ACTA1*, *ALG2*, *ANO5*, *ATP2A1*, *B3GNT1*, *BAG3*, *BIN1*, *CACNA1S*, *CAPN3*, *CAV3*, *CFL2*, *CHKB*, *CLCN1*, *CNTN1*, *COL12A1*, *COL6A1*, *COL6A2*, *COL6A3*, *CRYAB*, *DAG1*, *DES*, *DMD*, *DNAJB6*, *DNM2*, *DPAGT1*, *DPM1*, *DPM3*, *DYSF*, *EMD*, *FHL1*, *FKRP*, *FKTN*, *FLNC*, *GAA*, *GFPT1*, *GNE*, *GYG1*, *ISCU*, *ISPD*, *ITGA7*, *KBTBD1*3, *LAMA2*, *LAMP2*, *LARGE*, *LDB3*, *LMNA*, *MEGF10*, *MTM1*, *MYF6*, *MYH2*, *MYH7*, *MYOT*, *MYPN*, *NEB*, *ORAI1*, *PHKA1*, *PLEC*, *PNPLA2*, *POMGNT1*, *POMT1*, *POMT2*, *PYGM*, *RYR1*, *SCN4A*, *SEPN1*, *SGCA*, *SGCB*, *SGCD*, *SGCG*, *SQSTM1*, *STIM1*, *SYNE1*, *SYNE2*, *TCAP*, *TMEM43*, *TNNI2*, *TNNT1*, *TPM2*, *TPM3*, *TRIM32*, *TTN*, *VCP*, *VMA21*.

**Supplementary Table 2**

Clinical, radiological, electrophysiological and histopathological findings of the 26 reported patients with *SCN4A*-associated congenital myasthenic/ myopathy syndromes

(Excel: Supplementary table 2_20230223.xlsx)
